# Sex‐specific plasticity and genotype × sex interactions for age and size of maturity in the sheepshead swordtail, *Xiphophorus birchmanni*

**DOI:** 10.1111/jeb.12814

**Published:** 2016-01-08

**Authors:** K. Boulton, G. G. Rosenthal, A. J. Grimmer, C. A. Walling, A. J. Wilson

**Affiliations:** ^1^ The Roslin Institute and Royal (Dick) School of Veterinary StudiesUniversity of EdinburghMidlothianUK; ^2^ Department of BiologyTexas A&M UniversityCollege StationTXUSA; ^3^ Centro de Investigaciones Científicas de las Huastecas ‘Aguazarca’Calnali, HidalgoMexico; ^4^ Centre for Ecology and Conservation, BiosciencesCollege of Life and Environmental SciencesUniversity of ExeterPenryn, CornwallUK; ^5^ Institute of Evolutionary BiologyUniversity of EdinburghEdinburghUK

**Keywords:** genetic correlation, life history, plasticity, quantitative genetics, sexual conflict, *Xiphophorus birchmanni*

## Abstract

Responses to sexually antagonistic selection are thought to be constrained by the shared genetic architecture of homologous male and female traits. Accordingly, adaptive sexual dimorphism depends on mechanisms such as genotype‐by‐sex interaction (G×S) and sex‐specific plasticity to alleviate this constraint. We tested these mechanisms in a population of *Xiphophorus birchmanni* (sheepshead swordtail), where the intensity of male competition is expected to mediate intersexual conflict over age and size at maturity. Combining quantitative genetics with density manipulations and analysis of sex ratio variation, we confirm that maturation traits are dimorphic and heritable, but also subject to large G×S. Although cross‐sex genetic correlations are close to zero, suggesting sex‐linked genes with important effects on growth and maturation are likely segregating in this population, we found less evidence of sex‐specific adaptive plasticity. At high density, there was a weak trend towards later and smaller maturation in both sexes. Effects of sex ratio were stronger and putatively adaptive in males but not in females. Males delay maturation in the presence of mature rivals, resulting in larger adult size with subsequent benefit to competitive ability. However, females also delay maturation in male‐biased groups, incurring a loss of reproductive lifespan without apparent benefit. Thus, in highly competitive environments, female fitness may be limited by the lack of sex‐specific plasticity. More generally, assuming that selection does act antagonistically on male and female maturation traits in the wild, our results demonstrate that genetic architecture of homologous traits can ease a major constraint on the evolution of adaptive dimorphism.

## Introduction

Sexual dimorphism arises because fitness is limited by different traits in females and males (Bateman, 1948). Whereas fecundity is typically limiting for females, male fitness more often depends on traits that determine mating opportunities within the context of sexual selection imposed by female mate choice and/or male–male competition (Andersson, 1994). An important consequence of this is that homologous traits in males and females can have very different sex‐specific optima (i.e. sexually antagonistic selection). In some cases, sexual antagonism can be fully resolved over evolutionary time by the evolution of sex‐limited traits. However, where this is not the case, the degree of sex‐specific adaptation depends on constraints arising from genetic architecture that is shared between the sexes (Lande, 1980; Fairbairn & Roff, 2006; Poissant *et al*., 2010). Here, we describe a study of two key life‐history traits – age and size at sexual maturation – in a poeciliid fish and evaluate the extent that shared genetic architecture has the potential to limit sex‐specific adaptation given an expectation of sexually antagonistic selection in the wild. Additionally, we ask whether sex differences in plastic responses to changing levels of conspecific competition offer an alternative route to sex‐specific adaptive phenotypic expression.

Tests of the hypothesis that shared genetic architecture constrains sex‐specific adaptation have focussed largely on estimating the cross‐sex genetic correlation (subsequently denoted *r*_MF_) for homologous traits expressed in males and females (Walling *et al*., 2014; e.g. Pavitt *et al*., 2014). Strong cross‐sex correlations, whether positive or negative, mean that sex‐specific homologous traits are not free to evolve independently of one another. In the situation where directional selection is antagonistic in the two sexes, the ability of each sex to reach its own optimum will be maximally constrained at *r*_MF_ = 1 (with no constraint when *r*_MF_ = 0). Meta‐analysis suggests *r*_MF_ is usually strongly positive but negatively correlated with the degree of sexual dimorphism and is lower for traits closely linked to fitness (Poissant *et al*., 2010). These patterns are consistent with the expectation that sex‐specific genetic architecture (and hence reduced *r*_MF_) will evolve to at least partially alleviate constraints on adaptive dimorphism (Charlesworth & Charlesworth, 1980). Nonetheless, sexually antagonistic selection persists despite dimorphism (Cox & Calsbeek, 2009), *r*_MF_ between homologous traits is typically positive (Poissant *et al*., 2010), whereas negative cross‐sex genetic correlations have been reported for fitness itself (Brommer *et al*., 2007). These observations suggest that constraints arising from shared genetic architecture between the sexes have not been fully resolved.

Although the patterns described above are consistent with the constraint hypothesis, the restricted focus on *r*_MF_ has been criticized as inadequate for understanding the potential for, and limitations to, sex‐specific adaption. There are several reasons for this. Firstly, evolutionary responses to sex‐specific selection on homologous traits will depend not just on the cross‐sex genetic correlation but also on the presence and form of genotype‐by‐sex (G×S) interactions more generally. Importantly, these can be manifest not just as *r*_MF_ < 1, but also as between‐sex differences in the levels of additive genetic variance (Walling *et al*., 2014; Wyman & Rowe, 2014). Secondly, although studies typically focus on the genetic evolution of (mean) sex‐specific traits, phenotypic plasticity can also contribute to sexual dimorphism (Stillwell *et al*., 2010). Not all plasticity is adaptive, and we also note the separation of genetic and environmental effects is not always clear‐cut (e.g. in the presence of genotype‐by‐environment interactions). However, where divergence of sex‐specific phenotypic optima is sensitive to local environmental parameters, decoupling of male and female plastic responses could be an important mechanism for allowing adaptive dimorphism (Hallsson & Bjorklund, 2012). For example, where male fitness is limited by intrasexual competition, delayed maturation can be advantageous for males, allowing avoidance of aggression from rivals until their chances of competitive success are improved, for example, at greater age and/or size (Studd & Robertson, 1985; Lyon & Montgomerie, 1986).

Here, we describe a study of sex‐specific maturation traits in *Xiphophorus birchmanni,* (Lechner and Radda, 1987, sheepshead swordtail) that examines the potential for cross‐sex genetic constraints and sex‐specific plasticity. Although quantitative genetic analyses of cross‐sex genetic architecture have not previously been conducted in this species, several lines of evidence from other *Xiphophorus* species suggest that sexually antagonistic selection is likely to occur in the wild and that adaptive dimorphism may be facilitated by both cross‐sex decoupling of genetic processes (manifest as G×S) and plastic responses to the social environment. For instance, male‐specific genetic variance for life‐history traits is known to arise from Y‐linked loci with major effects on maturation age and size in populations of *X. maculatus* (platyfish; Schreibman & Kallman, 1977; Basolo, 1988) and northern swordtails (*X. nigrensis*,* X. montezumae*,* X. multilineatus*; Kallman, 1983; Ryan *et al*., 1992; Lampert *et al*., 2010). Nonetheless, behavioural and ecological studies indicate intersexual conflict is often ongoing and likely to be contingent on the social environment (Campton & Gall, 1988; Walling *et al*., 2007). Growth in male swordtails is almost determinate (i.e. slows down markedly at maturation) whereas females display indeterminate (continuous) growth (Evans *et al*., 2011). Size is important for both sexes, predicting dominance in males, who are territorial and compete agonistically over access to females (Prenter *et al*., 2008; Wilson *et al*., 2013) and fecundity in females. This can result in sex‐specific selection in certain environments, with males delaying maturation in the presence of other males to allow increased size (and thus expected dominance) at maturation, whereas females may even accelerate maturation under such circumstances (Borowsky, 1973; Walling *et al*., 2007). This illustrates the potential for both ongoing sexual conflict and contrasting sex‐specific plastic responses to contribute to resolution.

We use quantitative genetic analyses and experimental manipulation of the competitive environment (housing density) to provide the first assessment of the cross‐sex genetic architecture for maturation traits in *X. birchmanni* and to examine sex‐specific plasticity. To investigate cross‐sex genetic architecture, we apply pedigree‐based animal models to characterize the genetic covariance structure within and across sexes for age and size at maturation. We first estimate the magnitude of genetic variation in sex‐specific life‐history traits before formally testing for G×S interactions. If present, G×S interactions generate sex‐specific genetic variance that would facilitate evolution towards divergent phenotypic optima in male and females if sexually antagonistic selection does indeed operate on these traits as thought. If absent, shared genetic architecture will constrain further evolution of sexual dimorphism. To examine sex‐specific plasticity, we combine a density treatment (low vs. high) with analysis of variation in sex ratio among mixed family groups of juveniles raised to maturation. In general, we expect high rearing density to increase social stress arising from competitive interactions, leading to increased age and/or decreased size at maturity in both sexes. However, we anticipate that maturation traits will respond to male–male competition by being elevated in male‐biased groups housed at high density. We therefore predict that, if sex‐specific adaptive plastic responses are possible, then (conditional on main treatment effect of density) males should mature later and at larger size where male–male competition is high. Conversely, females should not delay maturation and may accelerate it (Walling *et al*., 2007).

## Materials and methods

### Fish husbandry and phenotyping

In the spring of 2010, one hundred adult *Xiphophorus birchmanni* (40 male, 60 female) were sampled by minnow trap from the Arroyo Coacuilco river (near Coacuilco, municipality of San Felipe Orizatlán, Hidalgo, Mexico) and imported to the UK (April 2011). They were housed in breeding groups comprising one male: three females, in 30‐L glass aquaria enriched with 3–5 mm diameter gravel and live plants. Water was maintained at 21–23 °C and a 12:12 hour light: dark cycle provided. Fish were fed twice daily on proprietary flake food (ZM foods, http://www.zmsystems.co.uk/) and previously frozen bloodworm and daphnia. Between August 2010 and May 2011, a captive bred generation was produced (*n* = 384) comprising 61 full‐sibling broods. Mean brood size born was 8.72 with a range from 1 to 24. Note that in some cases, multiple broods were collected from the same parental pair such that full‐sibship sizes represented in the data set are larger (mean = 16.18, range 1–51). Given the group housing regime, full‐sib families are nested within half‐sibships, with a total of 32 female and 19 male parents contributing to the offspring generation.

To collect broods, breeding groups were inspected daily and obviously gravid females were removed to isolation tanks enriched with stones and artificial plants to provide refuge for newborn offspring. Isolated females were also checked daily and returned to their breeding group tanks after giving birth. Broods were initially raised in 30‐L tanks, partitioned into two equal volumes (using an acrylic frame covered with fine‐gauge black nylon net) such that two families were raised in each tank. Tanks were grouped in ‘stacks’, each comprising six 30‐L tanks on a common recirculating water supply. This reduces the potential for between‐tank variation in water quality to introduce bias in genetic parameter estimation. Large broods were divided across tank partitions (setting a maximum of eight offspring per 15 L volume).

At an average age of 16 weeks (range 12–27 weeks), offspring were tagged with visible implant elastomer (http://www.nmt.us/products/vie/vie.shtml) and assigned to mixed family groups (*n* = 8 fish per group) subject to one of two density treatments. Low‐density groups (L) were housed in a full 30‐L tank, whereas high‐density groups (H) were housed in a half tank (i.e. 15 L volume partition of a 30‐L tank as described above). Six stacks, each comprising four low‐ and four high‐density groups on a recirculating water supply, were sequentially established (Fig. S1). Variation in age of fish entering the experiment was thus unavoidable as a stack could only be set up when 64 fish (eight groups × eight fish per group) reached a size suitable for tagging. Juveniles of this species cannot be sexed from external characters and the sex ratio of mixed family groups was therefore uncontrolled. Fish were fed twice daily with a mixed diet of fresh brine shrimp nauplii and crushed flake from birth until mixing of families and subsequently on the same diet as the wild caught breeding groups (with L and H groups receiving equal ration).

As part of a wider study involving long‐term behavioural and growth phenotyping (see Boulton *et al*., 2014), all fish in the experiment were measured for standard length (SL) using digital callipers and live weight (WT) using a digital balance at 4 week intervals, for a period of 28 weeks in total. Age of maturation (AM) was recorded for each individual as age at the first sample date where sexing from external morphology was possible. For males, this was when the first thickening of the anal fin rays associated with gonopodium formation became apparent (i.e. following Snelson, 1989). Typically, this is sooner than the development of other secondary male characters such as the nuchal hump, vertical stripes and enlarged and pigmented dorsal fin seen in this species. Female AM was determined from a suite of characters that differentiate juveniles from mature females (abdomen shape, darkening of the ‘gravid spot’ and lateral line). Given the lack of a single objective criterion to discriminate mature females from (unsexable) juveniles, measurement error is likely to be higher for female relative to male traits. However, all designations were made by a single investigator and were blind with respect to pedigree (and previously assigned sex) thus no expectation of bias arises with respect to genetic hypotheses (but see later Discussion). Weight and standard length at maturity (WTM, SLM) were simply defined as the corresponding size measurements at AM. Sex could not be determined for a total of ten fish still alive at the end of the 28‐week density treatment (*n* = 368 of the starting 384). However, continued monitoring of individuals for purposes out‐with this study (Boulton *et al*., 2014) meant that maturation trait data were subsequently obtained (one female and nine males).

### Analysis and quantitative genetic modelling

Exploratory data analysis was first conducted in R. We used simple linear models (i.e. without random effects) to estimate the relationships between maturation traits and to test for differences in phenotypic means across sex and density treatment classes. We then used a series of animal models fitted using asreml (Version 3; VSN International Ltd, Hemel Hempstead, UK) to formally test hypothesized plastic and genetic influences simultaneously as follows. First, for each sex‐specific maturation trait, we fitted a univariate model with the phenotype (*y*) of each individual (*i*) specified as follows:

(1) $$\begin{array}{cc} y_{i}\;= & \;\mu \;+\;\mathrm{Stack}\;+\;\mathrm{Density}\;+\;{\mathrm{GS}}_{i}\;+\;{\mathrm{SR}}_{i}\;+\\ & \;\mathrm{Density}:\mathrm{GS}\;+\;\mathrm{Density}:\mathrm{SR}\;+\;a_{i}\;+\;\varepsilon_{i} \end{array}$$

where *μ* is the mean, Stack is a seven‐level factor included to account for effects of any variation in water chemistry, and Density is a factor denoting treatment (Low (L) = 8 fish in 30 L, High(H) = 8 fish in 15 L). Group size (GS) and sex ratio (SR) experienced were defined as individual, rather than group‐level covariates. GS_i_ is the geometric mean number of fish in i's group, averaged across the monthly assay points up to and including age of maturity (AM_i_). Group size (and its interaction with Density) was included to control for any effects of mortality on phenotypes of surviving group mates (although, in practice, mortality levels were low; see Results). SR_i_ was similarly defined as the geometric mean (over assay points up to and including AM_i_) of the proportion of that individual's tank mates that are mature males [i.e. number of males in group excluding self/(number in group −1)]. Geometric means across assay points were used to define GS_i_ and SR_i_ to better capture cumulative effects of social environment, but both variables were then centred to an (arithmetic) mean of zero across all individuals to aid interpretation of model estimates.

Additive genetic merit (a_i_) was included as a random effect, assumed to be normally distributed with a mean of zero, and variance (*V*_A_, the additive genetic variance) to be estimated using the pedigree structure (Wilson *et al*., 2010). Residuals (Ɛ_i_) are assumed to be uncorrelated across observations and normally distributed with a mean of zero and variance (*V*_R_) to be estimated. Inference on fixed effects was based on conditional Wald *F*‐tests implemented by ASReml. Significance of *V*_A_ was determined by likelihood ratio tests (LRT) comparing model fit with and without the additive genetic effect. For testing *V*_A_ in univariate models, we assume the LRT test statistic is distributed as a 50:50 mix of $\chi_{1}^{2}$ and $\chi_{0}^{2}$ following (Visscher, 2006). Heritability was estimated as *V*_A_/*V*_P_ with the phenotypic variance (*V*_P_) determined as *V*_A_+*V*_R_ (i.e. conditional on fixed effects).

The univariate model was then extended to the multivariate case to estimate the genetic variance–covariance matrix (**G**) between all six sex‐specific traits (AM_F_, WTM_F_, SLM_F_, AM_M_, WTM_M_ and SLM_M_), with additive covariance estimates also rescaled to give the corresponding genetic correlations (*r*_G_). Fixed effects on each trait were as specified above. The full estimate of **G** was used to qualitatively assess the presence of G×S interactions. For more formal inference, we tested these conditions using a series of bivariate model comparisons applied to each homologous trait pair using likelihood ratio tests (Table 1). These comparisons tested for (A) heterogeneity of total phenotypic variance (*V*_P_) across sexes and (B) G×S interactions (manifest as *r*_MF_ < 1 and/or *V*_A(F)_ ≠ *V*_A(M)_). Note that in the absence of G×S, a_iF_ = a_iM_ for any pair of sex‐specific homologous traits (e.g. AM_F_, AM_M_), thus it follows that *V*_A(F)_ = *V*_A(M)_ and *r*_MF_ = 1 (the ‘No G×S’ scenario in Table 1). To further explore whether patterns of G×S detected were driven by cross‐sex genetic correlations or heterogeneity of *V*_A_ we compared (C) the full G×S model to one with freely estimated *r*_MF_ but with *V*_A(F)_ constrained to equal *V*_A(M)_ and (D) the full G×S model to one where *V*_A(F)_ and *V*_A(M)_ were free to differ but r_MF_ was constrained to equal +1. Note that for comparisons (B)–(D), all models included heterogeneous residual variance (i.e. *V*_R(F)_ and *V*_R(M)_ were free to differ) to prevent differences in environmental variance (or measurement error) generating spurious support for differences in sex‐specific additive variance estimates.

**Table 1:** Cross‐sex tests for (A) heterogeneity of phenotypic variance (*V*_P_) and (B) G×S interactions. Also presented are comparisons of the full G×S model to restricted scenarios where (C) _mf_ is freely estimated but *V*_A_ assumed homogeneous and (D) *V*_A(F)_ are allowed to differ *V*_A(M)_ but _mf_ is constrained to unity. For each comparison, null (H0) and alternate (H1) hypotheses are shown with statistical inference from likelihood ratio tests

Trait	Comparison	H0	H1	χ^2^	d.f.	*P*
AM	(A)	Homogeneous *V*_P_	Heterogeneous *V*_P_	3.80	1	0.051
	(B)	No G×S	G×S	5.90	2	0.052
	(C)	G×S	*r*_MF_ = +1, *V*_A_ assumed homogeneous	0.90	1	0.343
	(D)	G×S	*V*_A_ heterogeneous, *r*_MF_ = +1 assumed	1.70	1	0.427
WTM	(A)	Homogeneous *V*_P_	Heterogeneous *V*_P_	11.9	1	<0.001
	(B)	No G×S	G×S	12.5	2	0.002
	(C)	G×S	*r*_MF_ = +1, *V*_A_ assumed homogeneous	0.04	1	0.842
	(D)	G×S	*V*_A_ heterogeneous, *r*_MF_ = +1 assumed	7.10	1	0.029
SLM	(A)	Homogeneous *V*_P_	Heterogeneous *V*_P_	0.222	1	0.638
	(B)	No G×S	G×S	14.9	2	<0.001
	(C)	G×S	*r*_MF_ = +1, *V*_A_ assumed homogeneous	0.122	1	0.727
	(D)	G×S	*V*_A_ heterogeneous, *r*_MF_ = +1 assumed	9.07	1	0.011

## Results

### Exploratory data analysis

**Figure 1:**
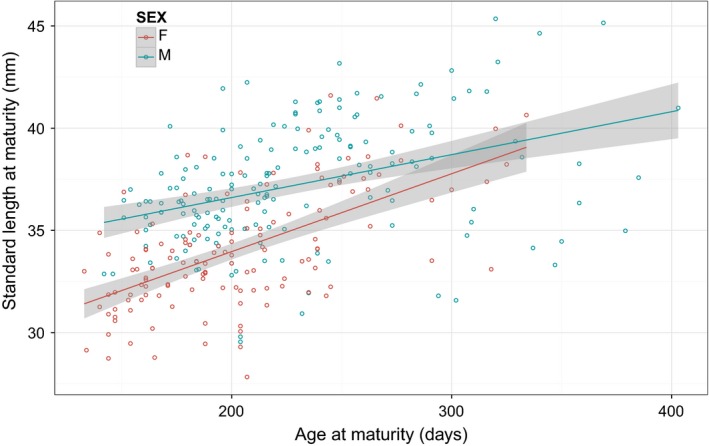
Observed size (standard length) at maturation as a function of age by sex. Circles denote phenotypic observations (*n*_F_ = 148, *n*_M_ = 195) whereas solid lines illustrate predictions from simple linear regressions with shaded areas denoting predicted mean ± SE.

Size at maturity increases with age at maturity as expected (Fig. 1). Regressions of size at maturity (SLM) on age at maturity (AM) are significantly positive in females (β (SE) = 0.034 (0.004) mm day^−1^, *P* < 0.001) and males (β (SE) = 0.019 (0.004) mm day^−1^, *P* < 0.001). Pooling data and including SEX (male relative to female) and SEX: AM effects (as well as a main effect of AM) in the linear model confirms that the relationship in females is significantly steeper (SEX: AM coefficient (SE) = −0.0153 (0.006), *t* = −2.705_339_, *P* = 0.007). The two measures of size at maturity are strongly correlated in both sexes (female, *r*_WTM.SLM_ = 0.953, *P* < 0.001; male, *r*_WTM.SLM_ = 0.919, *P* < 0.001); therefore, regressions of weight at maturity (WTM) on AM yield very similar patterns (results not shown). In addition to having steeper regressions of size on AM (Fig. 1), estimated correlations are stronger in females [*r*_AM.WTM_ = 0.554 (0.057), *r*_AM.SLM_ = 0.570 (0.056)] than in males [*r*_AM.WTM_ = 0.223 (0.068), *r*_AM.SLM_ = 0.350 (0.063)]. Testing against a null model of equal sex‐specific correlations indicates this difference is statistically significant for *r*_AM.WTM_ ($\chi_{1}^{2}$ = 13.1, *P* < 0.001) and r_AM.SLM_ ($\chi_{1}^{2}$ = 6.62, *P* = 0.010). While suggesting a degree of decoupling of size and age of maturity in males relative to females, we note that this result could also be driven by measurement bias (e.g. if body size unintentionally influences scoring of maturity status in females).

**Figure 2:**
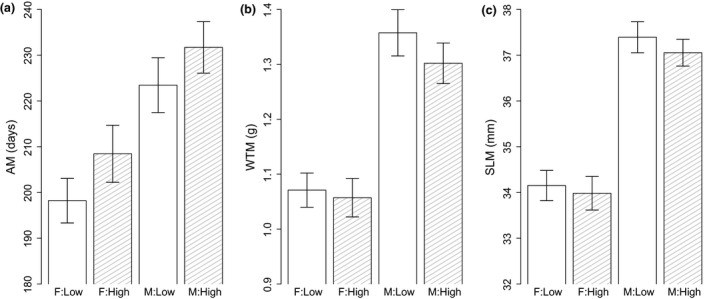
Mean observed (a) maturation age (AM), (b) weight (MWT) and (c) standard length (MSL) by sex for low‐ and high‐density treatments. White columns denote low (L)‐density treatment and shaded columns indicate high (H)‐density treatment for male (M) and female (F) values with bars showing mean ± SE (*n*_FL_ = 83, *n*_ML_ = 92, and *n*_FH_ = 65, *n*_MH_ = 103).

Comparison of trait means across sexes and treatment classes shows all traits to be sexually dimorphic but provides little evidence for plastic responses to the density treatment (Fig. 2). Note that as maturity status is assessed from external morphology using sex‐specific criteria here, we cannot be certain whether similar patterns would be found using physiological assays of maturation. However, based on criteria used, males mature on average 23.7 (5.60) days later than females (*t* = 4.22_341_, *P* < 0.001), at 0.248 (0.035) g heavier (*t* = 7.031_341_, *P* < 0.001) and at 2.99 (0.314) mm longer (*t* = 9.545_341_, *P* < 0.001; results from linear models with sex as categorical predictor). Fish tended to mature later and at smaller size at high density in both sexes but effects were largely nonsignificant. Overall mean AM is significantly higher at high density (linear model with Density as categorical predictor; difference of +12.4 (5.65) days, *t* = 2.196_341_, *P* = 0.029). Statistical support for this effect is not robust to the addition of the sex effect into the linear model although the effect size is similar (effect of high density = +10.4 (5.55) days, *t* = 1.878_340_, *P* = 0.061).

### Genetic variation and G×S interactions

Univariate animal models also provided evidence of genetic variation for maturation traits (Table 2). Heritability estimates from univariate models range from 0.113 to 0.462 and are significant at *α* = 0.05 except for male AM (h^2^ = 0.113 (0.112), $\chi_{0,1}^{2}$ = 2.11, *P* = 0.073). The corresponding estimates from the full (six trait) multivariate model are similar, although slightly higher (ranging from 0.166 to 0.477; Table 3). In general, genetic correlation estimates are characterized by high uncertainty, although the strong positive estimates between WTM_F_ and SLM_F_, and WTM_M_ and SLM_M_ are nominally significant based on |*r*_G_| > 1.96*SE. Of particular note are the cross‐sex (within‐trait) genetic correlation estimates (*r*_MF_) of 0.066, −0.291 and −0.108 for AM, WTM and SLM, respectively (Table 3). Thus, not only are cross‐sex genetic correlations not close to +1 (the expected value in the absence of G×S), but for size at maturity traits, they are actually negative (albeit not significantly less than zero).

**Table 2:** Estimated variance components and heritabilities (h^2^) from univariate animal models for age (AM), weight (WTM) and size (SLM) at maturity where *V*_P_, *V*_A_ and *V*_R_ are the phenotypic, additive genetic and residual variances, respectively

Sex	Trait	*V*_P_ (SE)	*V*_A_ (SE)	*V*_R_ (SE)	h^2^	χ^2^	*P*
Female	AM	1313 (185)	543 (311)	770 (229)	0.413 (0.202)	9.77	0.001
	WTM	0.067 (0.009)	0.024 (0.015)	0.043 (0.012)	0.360 (0.198)	6.20	0.006
	SLM	7.28 (1.05)	3.36 (1.83)	3.92 (1.30)	0.462 (0.208)	9.59	0.001
Male	AM	1764 (189)	200 (204)	1564 (227)	0.113 (0.112)	2.11	0.073
	WTM	0.115 (0.013)	0.028 (0.018)	0.087 (0.016)	0.242 (0.145)	7.40	0.003
	SLM	7.74 (0.931)	2.43 (1.45)	5.31 (1.11)	0.314 (0.166)	8.56	0.002

Also presented are likelihood ratio tests of *V*_A_ with the test statistics assumed to be distributed as a 50:50 mix of χ^2^ on 1 and 0 degrees of freedom are indicated. Standard errors are indicated in parentheses.

**Table 3:** Estimated heritabilities (shaded column) and genetic variance–covariance–correlation **(G** matrix) containing additive genetic variances (*V*_A_, shaded diagonal), covariances (cov_A_, below diagonal) and correlations (*r*_G_, above diagonal), all with standard errors indicated in parentheses. All parameter estimates are from a multivariate (six trait) model and are conditional on fixed effects fitted as described in main text

	Heritability	**G** matrix					
		AM_F_	WTM_F_	SLM_F_	AM_M_	WTM_M_	SLM_M_
AM_F_	0.477 (0.207)	643 (341)	0.410 (0.330)	0.361 (0.318)	0.066 (0.488)	−0.137 (0.406)	−0.378 (0.347)
WTM_F_	0.368 (0.202)	1.63 (1.79)	0.020 (0.020)	0.987 (0.025)	−0.410 (0.497)	−0.291 (0.436)	−0.084 (0.411)
SLM_F_	0.460 (0.208)	16.7 (19.3)	0.282 (0.165)	3.33 (1.82)	−0.526 (0.438)	−0.304 (0.394)	−0.108 (0.381)
AM_M_	0.166 (0.132)	28.8 (212)	−1.10 (1.43)	−16.5 (15.5)	294 (247)	−0.027 (0.518)	−0.029 (0.499)
WTM_M_	0.274 (0.154)	−0.619 (1.86)	−0.008 (0.012)	−0.099 (0.134)	0.083 (1.58)	0.032 (0.020)	0.892 (0.087)
SLM_M_	0.336 (0.165)	−15.5 (16.0)	−0.021 (0.104)	−0.319 (1.13)	−0.796 (13.7)	0.262 (0.147)	2.62 (1.48)

More formal comparison of bivariate (cross‐sex models) indicated that the null hypothesis of homogeneity in total phenotypic variance could be rejected for WTM (comparison (A) in Table 1). Statistical support for heterogeneous *V*_P_ in AM was marginally nonsignificant. In both cases, phenotypic variance conditional on fixed effects is higher in males (as is also qualitatively the case for SLM). The full G×S model (allowing *r*_MF_ < +1 and *V*_A(F)_ ≠ *V*_A(M)_) was significantly better than the null model for WTM and SLM (comparison (B) in Table 1) although marginally nonsignificant for AM ($\chi_{2}^{2}$ = 5.90, *P* = 0.052). The full G×S model was not significantly better than the more restricted formulation where r_MF_ was free but homogeneity of *V*_A_ imposed in any case (comparison (C) in Table 1) for any trait. However, it was preferred in comparison (D) for WTM and SLM. We therefore conclude that, for WTM and SLM, there is evidence for significant genotype‐by‐sex interactions driven primarily by cross‐sex genetic correlations of <1 (rather than heterogeneity of sex‐specific genetic variances). For AM [where *r*_MF_ = 0.066 (0.488)], statistical support for G×S is slightly more equivocal since, as noted above, the overall test for G×S was marginally nonsignificant. However, *post hoc* comparison between a model with no G×S (such that *V*_A(F)_ = *V*_A(M)_ and *r*_MF_ = ±1) and one where r_MF_ was allowed to depart from unity (with homogeneity of *V*_A_ imposed) suggests the latter is a significantly better fit to the data +1 ($\chi_{1}^{2}$ = 5.00, *P* = 0.025; comparison not shown in Table 1).

**Figure 3:**
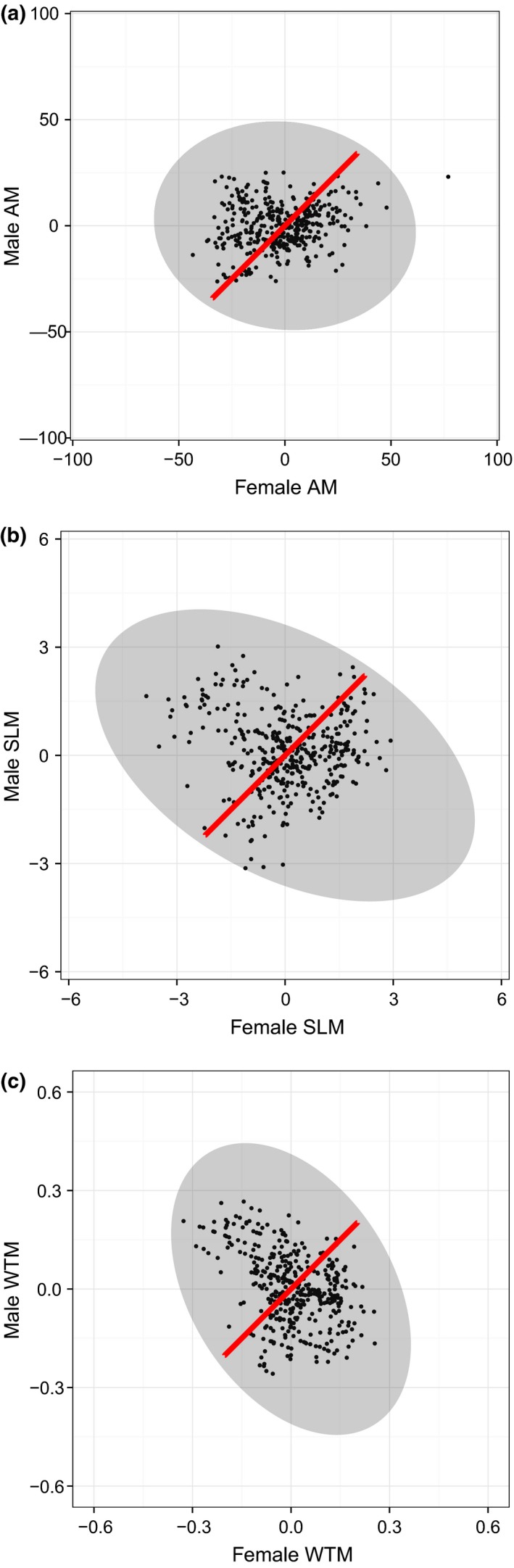
Cross‐sex genetic covariance structures for (a) maturation age (AM), (b) size at maturity (SLM) and (c) weight at maturity (WTM). Shaded ellipses denote the 95% confidence interval for the distribution of (bivariate) genetic merits in the population, and solid points indicate BLUP for individuals in the data (both from the full multivariate model). For comparison, red lines indicate the distribution of breeding values estimated under the assumption of no G×S.

A graphical representation of these G×S interactions is illustrated in Fig. 3, with the red line denoting the 95% confidence interval for the null distribution of bivariate breeding values (estimated assuming a_F_ = a_M_ such that *V*_A(F)_ = *V*_A(M)_ and *r*_MF_ = +1). In all cases, this line is a very poor fit to the distribution of bivariate breeding values estimated under the unconstrained model allowing G×S, represented by the grey ellipse.

### Animal model‐based estimates of phenotypic plasticity

Univariate animal models of sex‐specific traits confirmed the finding from our exploratory analysis that plastic responses to density were limited (Table 4). In males, a significant density by group size (GS) interaction was found on age of maturation (AM). The positive sign of this coefficient implies that the effect of higher GS (a significant reduction in male maturation age) is less strong at high density than at low (Table 4). For females, higher GS was associated with later maturation. No other effects of the density treatment were detected while GS did not significantly influence maturation size traits in either sex. Plastic responses to sex ratio variation were detected in both sexes (Table 4). For focal males, the presence of mature male group mates results in later maturity at larger size. For focal females, AM also increases with sex ratio (SR), but no significant effects on size at maturity were detected. Significant (or marginally nonsignificant) stack effects were found on all traits except male SLM (Table 4). These likely reflect average plasticity in response to between‐stack variation in water conditions and/or uncontrolled temporal patterns in the laboratory environment (as stacks were set up sequentially; see Materials and methods). As they are not relevant to hypotheses being tested here, we do not discuss these further.

**Table 4:** Fixed effect results from univariate animal models for age (AM), weight (WTM) and size at maturity (SLM), where GS is group size and SR is sex ratio

Trait	Effect	(Level)	Female				Male			
			Coefficient (SE)	d.f.	*F*	*P*	Coefficient (SE)	d.f.	*F*	*P*
AM	μ		236 (8.89)	1,14.2	1511	<0.001	267 (9.92)	1,11	3191	<0.001
	Stack	(B)	−33.0 (10.3)	5,124.6	8.10	<0.001	−31.3 (12.3)	5,120	4.65	<0.001
		(D)	−57.4 (10.0)				−40.2 (11.8)			
		(E)	−59.3 (11.4)				−34.9 (11.9)			
		(F)	−51.8 (13.3)				−57.0 (12.4)			
		(G)	−34.2 (11.5)				−37.4 (11.5)			
	Density	(High)	8.87 (6.74)	1,126.2	0.77	0.384	−8.89 (6.14)	1,175.1	2.19	0.144
	GS		21.5 (57.3)	1,132.1	5.15	0.026	−159 (29.1)	1,183.6	40.9	<0.001
	SR		70.0 (21.9)	1,135.9	18.5	<0.001	99.7 (21.6)	1,182.4	44.7	<0.001
	Density:GS		−84.7 (61.0)	1,120.3	1.93	0.170	87.7 (32.5)	1,176.8	7.30	0.008
	Density:SR		39.9 (37.0)	1,131.4	1.16	0.285	11.2 (27.3)	1,181.7	0.17	0.676
WTM	μ		1.08 (0.063)	1,13.3	846	<0.001	1.46 (0.082)	1,13.8	1149	<0.001
	Stack	(B)	0.093 (0.074)	5,121.8	2.25	0.054	0.020 (0.101)	5,134.2	3.95	0.002
		(D)	−0.067 (0.072)				0.002 (0.097)			
		(E)	−0.099 (0.082)				−0.042 (0.098)			
		(F)	0.147 (0.095)				−0.218 (0.102)			
		(G)	0.048 (0.083)				−0.316 (0.094)			
	Density	(High)	−0.029 (0.045)	1,126.6	0.39	0.533	−0.087 (0.048)	1,173.3	3.18	0.078
	GS		−0.186 (0.416)	1,132.6	0.78	0.380	0.285 (0.230)	1,183	1.10	0.298
	SR		0.134 (0.158)	1,136.1	0.93	0.338	0.636 (0.170)	1,179.6	22.3	<0.001
	Density:GS		0.048 (0.443)	1,120.8	0.01	0.908	−0.214 (0.255)	1,176	0.70	0.403
	Density:SR		−0.005 (0.268)	1,132.1	0.00	0.983	−0.088 (0.216)	1,178.6	0.17	0.678
SLM	μ		34.3 (0.664)	1,14.6	7039	<0.001	37.7 (0.678)	1,13.8	11733	<0.001
	Stack	(B)	0.691 (0.765)	5,126.5	2.23	0.056	0.566 (0.838)	5,137.5	1.29	0.272
		(D)	−1.04 (0.746)				0.109 (0.802)			
		(E)	−0.949 (0.842)				−0.151 (0.806)			
		(F)	1.24 (0.981)				−1.10 (0.844)			
		(G)	0.883 (0.852)				−1.14 (0.770)			
	Density	(High)	−0.336 (0.495)	1,125.4	0.75	0.388	−0.668 (0.387)	1,171.6	2.94	0.091
	GS		−0.593 (4.21)	1,131.4	0.65	0.421	2.23 (1.85)	1,181.3	0.11	0.731
	SR		2.20 (1.61)	1,135.5	2.87	0.095	5.41 (1.37)	1,177.6	26.0	<0.001
	Density:GS		−0.875 (4.48)	1,119.4	0.04	0.839	−2.45 (2.05)	1,174.9	1.42	0.237
	Density:SR		0.594 (2.72)	1,130.5	0.05	0.821	−0.531 (1.73)	1,176.4	0.09	0.753

## Discussion

Our results demonstrate that different genetic mechanisms underlie variation in age and size at maturity in males and females. The genetic architecture therefore acts to mitigate intersexual conflict over these traits. This finding is consistent with previous studies in swordtails that show a small number of loci on the Y chromosome can be responsible for much of the among‐male variation in these traits. Sexual dimorphism is evident in all traits, with males tending to mature later and at larger size (based on our assessment of maturity status), and in the relationship between age and size at maturity. Although positively correlated in both sexes (as expected from studies of other fish including poeciliids (Snelson, 1984; Rowe & Thorpe, 1990; Morita & Fukuwaka, 2006), AM explains more variation in maturation size traits for females than males. Regressions of size traits on AM also show that absolute juvenile growth is faster in females. However, despite this sexual dimorphism in mean phenotype, we found only limited support for putatively adaptive sex‐specific plastic responses in maturation traits to competition (i.e. density, sex ratio). In what follows, we first highlight the evolutionary implications of the G×S effects found before considering the results pertaining to plasticity in more detail.

### Genetic (co)variance structure and G×S interactions

Quantitative genetic models revealed the presence of both genetic (co)variation and significant genotype‐by‐sex interactions. Thus, not only are life histories free to evolve, but also to the extent that natural selection acts antagonistically in the wild, there is also potential for adaptive evolution of increased sexual dimorphism. Estimates of heritability were lower in males than in homologous female traits, a pattern driven by higher levels of residual variation rather than differences in additive genetic variance (*V*_A_; discussed further below). Competition in general, and contest competition in particular, is expected to increase variance in resource dependent traits, as winners gain resource at the expense of losers (Wilson, 2014). Thus, higher residual variance in male traits is consistent with the well‐documented importance of male–male competition in swordtails (Earley, 2006), including *X. birchmanni* (Wilson *et al*., 2013). Within‐sex genetic correlations (*r*_G_) between SLM and WTM were close to +1 but interestingly we did not find strong genetic correlations between these traits and age at maturity. In females, moderate positive (but nonsignificant) estimates of *r*_G_ were found between traits, whereas in males, these estimates were close to zero. Within both sexes, estimates of *r*_G_ between traits were characterized by high uncertainty and therefore should be interpreted cautiously. Nonetheless, while there is perhaps some suggestion that the tighter (positive) phenotypic correlation between age and size of maturity in females relative to males is mirrored at the genetic level, there is no strong evidence for a genetic basis to the widely assumed fitness trade‐off between age and size of maturity in either sex (Stearns, 1992; Roff, 2002; Kruuk *et al*., 2008).

Formal testing for G×S interactions provided evidence that the genetic basis of life‐history variation differs between males and females. While *V*_A_ for homologous traits did not differ significantly between the sexes as has been reported elsewhere (Wyman & Rowe, 2014), estimated cross‐sex genetic correlations were close to zero, in contrast to the vast majority of empirical studies of *r*_MF_. These low genetic correlations between homologous traits in males and females imply a considerable degree of genetic decoupling. Consequently, additive variance can be considered largely sex‐specific and shared genetic architecture is not expected to be an important constraint on adaptive dimorphism if males and females are subject to antagonistic selection. While our experiment does not provide any information on the detail of this genetic architecture, low estimates of *r*_MF_ will arise from sex‐linkage and/or sex‐limited expression of autosomal genes. Both phenomena represent evolutionary solutions to the problem of sexual antagonism (Charlesworth & Charlesworth, 1980) that are known to affect expression of size, growth, colouration and behavioural traits in poeciliids (Lindholm & Breden, 2002; Postma *et al*., 2011). Y‐linked variation with allelic effect sizes sufficiently large to induce phenotypically distinct male morphs are known in some *Xiphophorus* species (Schreibman & Kallman, 1977; Ryan *et al*., 1992; Cummings & Gelineau‐Kattner, 2009). For instance, in *X. nigrensis* and *X. multilineatus* membership of one of three adult size morphs is strongly predicted by copy number variation of the melanocortin 4 receptor (mc4r) gene on the Y chromosome (Lampert *et al*., 2010). Although there is no evidence of distinct male size morphs, in *X. birchmanni,* we note that higher allelic copy numbers (and or variation in copy number) could easily rise to unimodal phenotypic distributions. Thus, we consider mc4r, a good candidate for contributing to the male‐specific genetic variance found here, although this remains to be tested.

Our conclusions with respect to the quantitative genetics of male and female life‐history traits are contingent on several potentially important caveats. Firstly, parameters are estimated under an additive model and assume absence of maternal and/or other early life common environmental effects (as offspring were raised in families until large enough to tag and mix). Maternal effects on offspring traits are known to occur in poeciliid fishes, including *X. birchmanni* (Reznick *et al*., 1996; Kindsvater *et al*., 2012), although the extent of their persistence to impact adult traits is variable (Lindholm *et al*., 2006). Here, the failure of some wild caught adults to reproduce under laboratory conditions meant the size and structure (i.e. limited half‐sib structuring) of our progeny data set is not sufficient to effectively disentangle any maternal effects. While upward bias of additive genetic parameters is certainly possible (Falconer & Mackay, 1996), no systematic bias towards finding reduced r_MF_ is expected. Secondly, although we have considered both age and size of maturation in this study, genetic covariance structure may well exist with other components of life history (e.g. fecundity, adult growth and longevity) within and between sexes. Multivariate analysis can identify constraints not apparent from pairwise genetic correlations alone (Walsh & Blows, 2009), although we note the converse is also true. Specifically, it has recently been argued that multivariate treatments of sexual dimorphism may actually reveal greater evolutionary potential for dimorphism than previously thought based on *r*_MF_ estimates (Wyman *et al*., 2013); see also (Gosden & Chenoweth, 2014; Walling *et al*., 2014) for related discussion. Thirdly, we have implicitly assumed an absence of GxE such that genetic covariance is modelled as being constant with social environment (i.e. density and/or sex ratio). Available data were insufficient to support modelling of life‐history traits disaggregated by both sex and environment, and thus, our genetic estimates should be viewed as averaged across any genotype‐by‐environment (GxE) effects present. GxE can be of considerable importance for sexually selected traits (Hunt & Hosken, 2014), although explicit studies of GxExS are currently lacking. Despite the formidable empirical challenges, we suggest that experiments to address this gap in our knowledge could offer great insights into the evolution of sexual dimorphism. This is because GxExS implies the presence of sex‐limited genetic variance, and thus potential for independent evolution, not just of male and female traits but also of male and female plasticity in those traits.

### Sex specificity of social plasticity

There was evidence of some social plasticity in both sexes, although life‐history traits were influenced more by sex ratio variation (SR) than by the experimentally applied density treatment. Broadly, responses are consistent with predictions made under the presumption that high density increases competition and that for males, this is exacerbated by a high sex ratio (i.e. the presence of more mature rivals). However, while both sexes show a similar trend towards later maturation at smaller size under high density, effects were small and not statistically supported in the mixed models. Nevertheless, to the extent that plastic responses to the treatment are occurring, the trend is consistent with negative density dependence on life history with respect to expected fitness consequences. Although we also found a significant positive effect of group size (GS) on female maturation age (consistent with density dependence), in males, the corresponding effect was actually negative (though less so at high density). These latter results are difficult to interpret as GS effects were modelled to control for within‐group mortality rather than to test *a priori* hypotheses. It is possible that agonistic interactions between males that have already matured within‐group increase their mortality risk, and thus, lower GS may indicate that competition has been intense for males. However, as only 16 of 384 fish died before the end of the experiment variation in GS is very low (and nonrandom with respect to groups). We therefore consider it quite possible that this result is an artefact arising from data structure.

The direct effects of density on life history were thus limited and also similar in males and females. However, we also predicted plasticity in response to sex ratio variation and sex differences in this response, with males responding to a greater extent at high density. Perhaps unsurprisingly given the lack of main effects, we found no significant interactions between density and sex ratio on either male or female life history to support the second of these predictions. Nevertheless, both male and female traits did respond to sex ratio, albeit in similar directions. Maturation occurs later and at larger size in the presence of more adult males in both sexes. This is consistent with our predictions of adaptive plasticity for males. Increased male–male competition results in sexual selection that favours larger maturing males in swordtails, even if this comes at the cost of a delayed maturation time (Basolo, 1988; Beaugrand *et al*., 1996; Benson & Basolo, 2006).

Delayed female maturation in male‐biased groups is counter to our expectations. With increasing numbers of mature males, we predicted females should not delay maturation and may even advance it. This prediction was based on an assumption that more available males would increase the fitness benefits of early maturation. It is possible that mature males were socially dominant to females and thus able to monopolize resources (e.g. food) in the experimental conditions. If so, delayed female maturation may be a consequence of resource limitation. Harassment by males could also be a factor as it is energetically costly for both sexes and can disrupt female social structures (Darden *et al*., 2009; Darden & Watts, 2012). Given the lack of fitness data and the presence of artificial conditions, we cannot completely exclude the possibility that this response confers some potential benefits under natural conditions. Nonetheless, while SR effects on female WTM and SLM are positive, they are modest and not significant. Consequently, it seems unlikely that delayed maturation in females can be compensated for by size‐related increases in fecundity later. Whatever the explanation, our results suggest that males and females tend towards later and larger maturity in the presence of mature male group mates, the response being larger in males, where we predicted it to be adaptive.

## Conclusions

In summary, our study sought to test for sex‐specific genetic and social environment effects on age and size of maturity in the sheepshead swordtail. We found that these traits are sexually dimorphic and responsive to social factors expected to determine the intensity of competition. At high density, there was a general tendency towards maturing later and at smaller size in both sexes. Although generally consistent with expected non‐sex ‐specific density dependence, these effects were modest and nonsignificant. Moreover, males were also found to delay maturation in the presence of mature rivals, a putatively adaptive response given that this results in larger adult size (and thus higher success in male–male competition). Interestingly, females showed a similar pattern (albeit with smaller phenotypic changes) and delayed maturation in response to increased sex ratio. This is contrary to adaptive predictions, suggesting that a lack of sex‐specific plasticity could limit the expression of (adaptive) sexual dimorphism in social environments where male–male competition is high. Conversely, our quantitative genetic analyses illustrate that life‐history traits are subject to G×S interactions – age and size at maturity are heritable in both sexes but the cross‐sex genetic correlations between homologous traits are close to zero (and significantly less than +1). Thus, to the extent that natural selection on maturation traits does act antagonistically in the wild, our results show that the genetic architecture of homologous traits can ease a major constraint on the evolution of adaptive dimorphism.

## Supporting information


**Figure S1** Schematic of recirculating stack system used to house fish.
