# Supplementary material for: Sex‐specific plasticity and genotype × sex interactions for age and size of maturity in the sheepshead swordtail, Xiphophorus birchmanni
Source: J Evol Biol. 2016 Jan 8;29(3):645–56. doi: 10.1111/jeb.12814 (PMC5102681; doi:10.1111/jeb.12814)
Supplement: Supplementary file 1 — Figure S1 Schematic of recirculating stack system used to house fish. [file JEB-29-645-s001.docx]

Fig. S1: Schematic of recirculating stack system used to house fish. Each stack comprised six tanks arranged into eight housing units (four full-size tanks = 30 L (low density), and four half-size tanks = 15 L (high density)). Filtered, heated water was pumped up from the sump, then recirculated through the stack under gravity (blue arrows indicate flow of water). Eight fish were allocated to each of the units that were enriched with pebbles and plants (not shown).

Filter media

Pump

Heater

1

2

3

4

5

8

6

7

Sump
